# Nanostructured TiO_2_ Carbon Paste Based Sensor for Determination of Methyldopa

**DOI:** 10.3390/ph11040099

**Published:** 2018-10-05

**Authors:** Luane Ferreira Garcia, Carlos Eduardo Peixoto da Cunha, Emily Kussmaul Gonçalves Moreno, Douglas Vieira Thomaz, Germán Sanz Lobón, Rafael Luque, Vernon Somerset, Eric de Souza Gil

**Affiliations:** 1 Faculdade de Farmácia, Universidade Federal de Goiás, Goiânia, GO 74690-970, Brazil; luane.fg@hotmail.com (L.F.G.); milykussmaul@gmail.com (E.K.G.M.), douglasvthomaz@gmail.com (D.V.T.), manger84@gmail.com (G.L.); 2 Departamento de Química Orgánica, Universidad de Cordoba, E14014 Cordoba, Spain; q62alsor@uco.es; 3 Peoples Friendship University of Russia (RUDN University), 6 Miklukho-Maklaya str., 117198 Moscow, Russia; 4 Department of Chemistry, Cape Peninsula University of Technology, Bellville 7535, South Africa; vsomerset@gmail.com

**Keywords:** pencil graphite, metal oxide nanostructures, low cost, phenolic drug

## Abstract

Methyldopa is a catecholamine widely used in the treatment of mild to moderate hypertension whose determination in pharmaceutical formulae is of upmost importance for dose precision. Henceforth, a low-cost carbon paste electrode (CPE) consisting of graphite powder obtained from a crushed pencil stick was herein modified with nanostructured TiO_2_ (TiO_2_@CPE) aiming for the detection of methyldopa in pharmaceutical samples. The TiO_2_-modified graphite powder was characterized by scanning electron microscopy and X-ray diffraction, which demonstrated the oxide nanostructured morphology. Results evidenced that sensitivity was nonetheless increased due to electro-catalytic effects promoted by metal modification, and linear response obtained by differential pulse voltammetry for the determination of methyldopa (pH = 5.0) was between 10–180 μmol/L (Limit of Detection = 1 μmol/L) with the TiO_2_@CPE sensor. Furthermore, the constructed sensor was successfully applied in the detection of methyldopa in pharmaceutical formulations and excipients promoted no interference, that indicates that the sensor herein developed is a cheap, reliable, and useful strategy to detect methyldopa in pharmaceutical samples, and may also be applicable in determinations of similar compounds.

## 1. Introduction

Hypertension is a disease whose insidious progression presents many threats to human health, which in turn led to the development of a wide therapeutic arsenal to minimize patient morbidity. Methyldopa [α-methyl-β-(3,4-dihydroxyphenylalanine)] is noteworthy amongst these drugs, as it is a phenolic derivative broadly prescribed to manage mild to moderate hypertension by relaxing blood vessels. Nonetheless, the implications that this drug has on therapeutics imply the importance of its determination in pharmaceutical samples. In this context, methyldopa intrinsic chemical features may be explored through myriad analytical principles in order to optimize its detection. Amongst these features, the hydroxyl groups present in the molecule may undergo oxidation, which allows henceforth, detection by electrochemical sensors [1,2,3,4,5]. 

Literature reveals that several analytical tools have been employed for the analysis of methyldopa. Some of the techniques used are predominantly based on spectrophotometry [6,7,8], high performance liquid chromatography [9,10,11], and electroanalysis [1,2,3,4,5]. The use of pharmaceutical electroanalysis for methyldopa detection has been the subject of several studies [1,2,3,4,5,6,7,8,9,10,11,12]. Indeed, the low consumption of reagents, cheapness of electrochemical apparatus, combined with good sensibility, broad applicability, and suitable selectivity are some of the main attractive features of this method. Moreover, both sensibility and selectivity can be improved by changes in electrode matrix composition [12,13,14,15,16,17,18,19,20,21].

Literature reports the use of several sensing materials in the determination of anti-hypertensive drugs such as methyldopa. Amongst these electrodic devices, the most used are boron doped diamond [22,23] and glassy carbon electrode (GCE) [21,22,23,24]. Albeit widely explored in analytical studies, diamond-based electrodes are unpractical due to their high cost, while GCE may not provide adequate reproducibility hence electrode surface fouling. In this context, carbon paste-based electrodes (CPE) are thriving in pharmaceutical analysis due their high versatility concerning modifications, easy preparation, low cost, and easy surface renewal [3,13,14,15,16,17,18,19,20,21]. 

CPEs obtainment is feasible through low-cost carbon-based materials such as pencil graphite, which can be modified with different materials, such as nanostructured metal oxides, which can be anchored on a carbon surface through a plethora of easily feasible chemo-adsorption methods, and henceforth improve sensibility through electro-catalytic effects. [2,3,13,14,15,16,17,18,19,20,21].

In view of the importance that anti-hypertensive drugs have in therapeutics, and the relevance of developing cheaper, faster, and simpler methods to determine their concentrations in pharmaceutical samples, this work reports the use of nanostructured metal oxides anchored on a carbon graphite surface in the development of a modified CPE to target methyldopa detection. The analytical performance of the nanosensor was herein investigated, focusing on the detection of a catechol probe and the analyte methyldopa.

## 2. Results

### 2.1. Graphite Characterization

**Figure 1:**
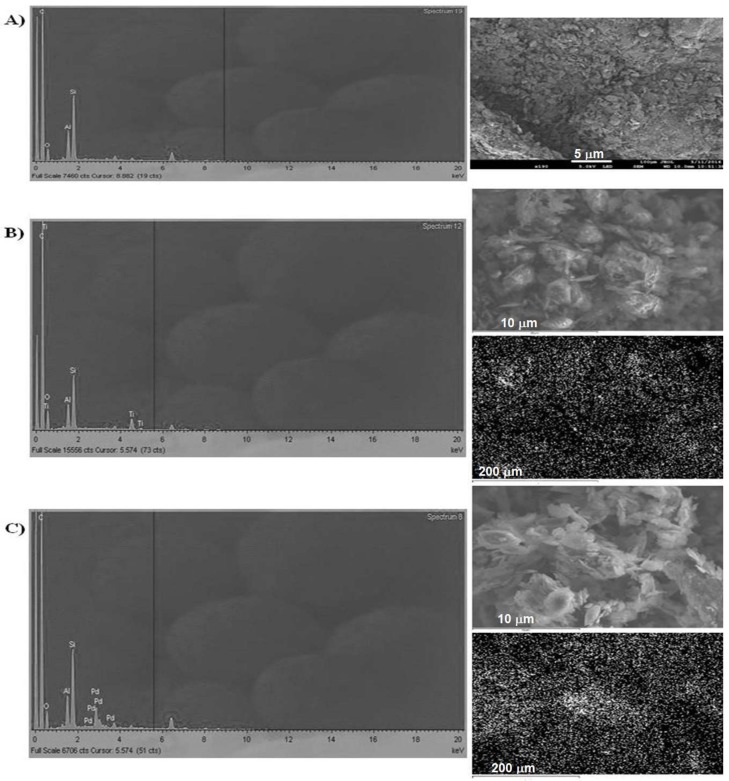
Results for SEM and energy-dispersive X-ray (EDX) characterization of: graphite powder (**A**), and carbon nano-modified powder with titanium (**B**) and palladium (**C**) oxide particles.

The results for the SEM and energy-dispersive X-ray (EDX) characterization of the graphite powder modified with palladium and titanium metallic particles are depicted in Figure 1 and Table 1.

The optimum percentage composition (from EDX analysis) of TiO_2_@C and PdO@C per CPE was 1.6% and 6%, respectively.

### 2.2. Electrochemical Impedance Spectroscopy

After the characterization outlined above, Pd and Ti oxide modified graphite materials were used to produce nanostructured-metal @CPE sensors.

**Figure 2:**
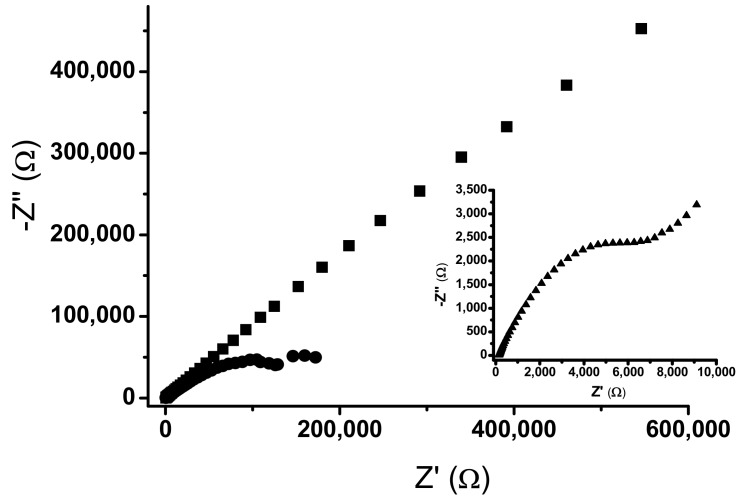
The Nyquist plots obtained for PdO@CPE (■) and CPE (●). Insert: Nyquist plot for TiO_2_@CPE (▲).

The new materials were electrochemically characterized by electrochemical impedance spectroscopy (EIS) analysis, since it was the most suitable technique to understand the electron transfer kinetics of the nano-modified graphite composition in the electrochemical cell considering the electrode surface area modification [14,15,17]. Results depicted in Figure 2 evidence the Nyquist plot obtained for the different sensors that were tested. In addition, the electric equivalent models were proposed for TiO_2_@CPE (Figure 2).

The data concerning the Randles equivalent circuit of each electrode was gathered and displayed in Table 2. The *Rs* is uncompensated ohmic resistance of electrolyte solution; *Rp* represents the polarization resistance and is related to electron transfer trough each electrode material; *C* is the pseudo-capacitance and frequency independent taken from the constant phase element, which describes the imperfect capacitive behavior of the double-layer; *Y* is the admittance.

### 2.3. Electrocatalytic Response

**Figure 3:**
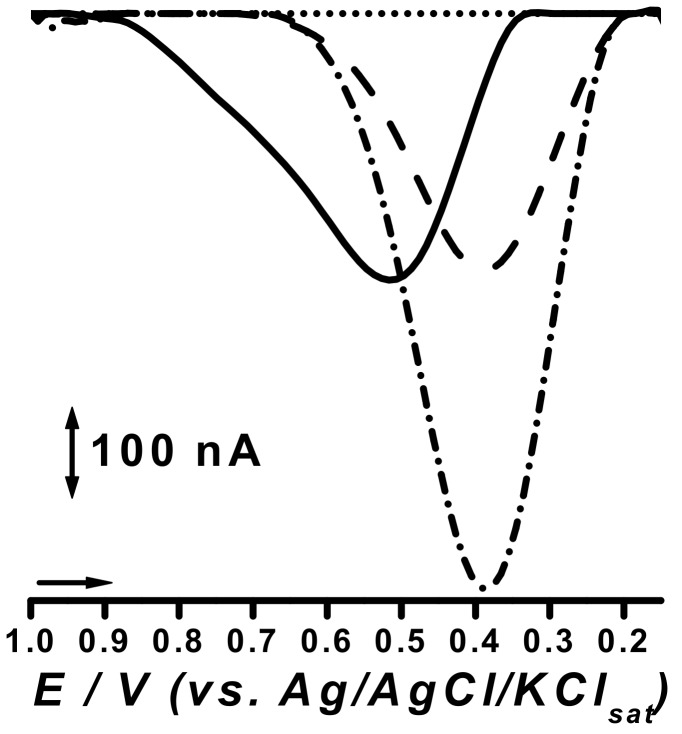
Differential pulse (DP) voltammograms obtained for 133 µM catechol solution at carbon paste electrode (CPE) (▪ ▪ ▪), PdO@CPE (▬▬), and TiO_2_@CPE (▪ • ▪ ) sensors and for blank at TiO_2_@CPE sensor (• • •), all in 0.1 M phosphate buffer solution (PBS), pH 7.0.

The optimized nano-modified graphite powder sensors were evaluated for phenol detection due to the structural similarities between this compound and methyldopa. Nonetheless, literature reports the study of potential electrocatalytic effects through differential pulse (DP) analysis on a phenolic probe such as catechol, which henceforth guided this study. Results were compared with that of the unmodified CPE sensor and are depicted below (Figure 3). 

### 2.4. Sensor Application in Methyldopa Detection

**Figure 4:**
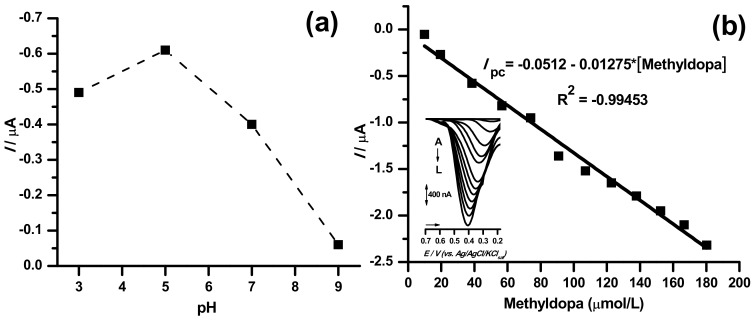
(**a**) pH response study for the TiO_2_@CPE sensor. (**b**) Calibration curve obtained from differential pulse voltammetry (DPV) analysis in 0.1 mol/L PBS (pH 5.0), for increasing concentrations (A) to (L) of methyldopa (see insert graph).

In order to evaluate the analytical performance of the TiO_2_@CPE sensor, the calibration graph was constructed for methyldopa in the respective optimum pH condition (Figure 4). 

The linear ranges from 10 to 180 µmol/L were obtained for methyldopa (*I_pc_* = −0.0512–0.01275*[methyldopa]; R^2^ = −0.99453) using TiO_2_@CPE sensor (Figure 4b). Thus, the calibration curve presented good linearity.

The analytical performances of the TiO_2_@CPE sensor were also compared to other systems described in the literature concerning methyldopa detection (Table 3). 

### 2.5. Sensor Applicability in Commercial Samples

The selectivity of the differential pulse voltammetry (DPV) method was assessed by evaluating the interference of high amounts of usual excipients on the analytical signal of pure standard solution. Results are depicted below (Table 4).

The nano-modified graphite powder analytical sensors developed in this study were further applied for the detection of methyldopa in pharmaceutical formulation (tablets), and the recovery was compared with the official method [25]. Results are depicted below (Table 5).

## 3. Discussion

Literature reports that electrodes modified with nanostructured oxides can increase the efficiency of drug detection in electroanalysis through electro-catalytic effects. These phenomena are usually associated to modifications with transition metal oxides, whose electron accepting properties may enhance analyte oxidation when anodic scans are performed [13,14,15,16,17,18,19,20,21]. 

According to the nature of each electrode modifier, i.e., metal oxide, different interactions will occur in the electrode surface area. SEM and EDX characterization in Figure 1 demonstrated that the PdO anchoring on graphite powder was more than three times higher than that of TiO_2_. However, the Pd deposition occurred in a less homogeneous way, which corroborates to punctual over-accumulation of metal particles. Therefore, despite the higher proportions of Pd at carbon surface, the heterogeneous dispersion compromised the nanostructured modification (Figure 1). These findings are nonetheless in consonance to previous works reported by our group, wherein Ti based modifications tend to homogeneously adsorb on carbon-based surfaces [26].

The SEM and EDX results show that deposition processes of both metal oxide nanoparticles at the smooth carbon surface occurred successfully, as compared to the parent carbonaceous material (Figure 1). Also, the metallic percentages in the nano-modified carbon powder ranged from 4.00% to 2.25% for Ti, and from 10.63% to 13.76% for Pd (Table 1). 

EIS results evidenced that TiO_2_@CPE presented the best electrodic features, as its Z” and Z’ axis impedance was nonetheless inferior to all other herein studied electrodes (Figure 2). According to the admittance values (*Y*) of ferrocyanide probe in its Warburg diffusion, TiO_2_@CPE promoted improvement in electron transfer kinetics over the unmodified form (CPE) (Table 2). Thus, the EIS study for TiO_2_@CPE showed an improvement in reaction kinetics. Moreover, TiO_2_CPE presented the highest pseudo-capacitance values amongst all studied electrodes, which may correlate to better electrodic properties. This finding is nonetheless corroborated by similar reports in literature, in which solid electrodes possessing optimal charge kinetics tend to exhibit higher capacitive behavior due to double layer formation [27,28,29].

Concerning sensibility, TiO_2_@CPE also presented the best features, as the electro-catalytic gain observed for the PdO@CPE sensor was null, with a negligible increase of peak currents when compared to unmodified CPE (Figure 3). Moreover, the cathodic peak potential, at *E_pc_* of 0.50 V (vs. Ag/AgCl), underwent a cathodic shift of 0.1 V, which in fact made this system more vulnerable to eventual interfering substances (Figure 3).

In turn, the TiO_2_@CPE sensor presented peak currents more than twice higher than those of the PdO@CPE sensor, which presented no peak potential shifting (Figure 3). Therefore, further assays were carried out only with the TiO_2_@CPE sensor. Nonetheless, similar results were obtained with perovskite-type LaFeO_3_ nanoparticles in sonogel platform [13], as well as with CuO modified CPE in phenolic compounds determination [16], and with a modified graphite/SrPdO_3_ electrode with gold nanoparticles for glucose determination [15].

The best response was observed at pH 5.0, as stated by the highest peak current values (Figure 4). Indeed, the higher aqueous solubility of methyldopa at mild acid pH and the involvement of proton on the redox process are crucial kinetic factors [18,21].

Analysis of the results depicted in Table 3 have shown that although the LOD for the TiO_2_@CPE sensor in this study was not as sensitive, when compared to other methyldopa sensors, a good comparable linear range was still obtained, and the application of this sensor in acidic media was found to be very favorable. Nonetheless, most studies used GCE modifications, which require strenuous electrode pre-preparation, meticulous surface polishing, and made use of high cost materials, while the work herein reported presented a CPE matrix of minimal cost and easy surface renewal. The voltammetric technique used in our work was also implicated in higher LOD, due to the elevated sensibility of square wave-based voltammetry, which was used in the reports cited in Table 3. However, we opted to use DPV due to the fact that it provides cleaner and more reproducible data.

The determination of methyldopa in tablets of 250 mg using the nano-modified graphite powder analytical sensor TiO_2_@CPE demonstrated a recovery of nearly 100%, and RSD lower than 5% (*n* = 3) (Table 4), thus evidencing the accuracy and precision of the modified electrode TiO_2_@CPE sensor. Furthermore, data showed that excipients do not promote any interference in the assays, which is nonetheless noteworthy for pharmaceutical analysis.

## 4. Materials and Methods 

### 4.1. Reagents, Samples, and Solutions

Electrolyte solutions were prepared using analytical grade reagents purchased from Vetec Química Fina Ltd. (Rio de Janeiro, Brazil), and diluted in purified water (conductivity ≤ 0.1 µS.cm^−1^) obtained from Milli-Q purification system Millipore S/A (Molsheim, France).

CPE was prepared using carbon powder and mineral oil (Sigma-Aldrich, St. Louis, MO, USA). The carbon powder was obtained by crushing Milan^®^ B 5.2 mm pencil for 7 minutes in a high energy planetary ball mill (Emax PM 100, Retsch^®^, Haan, Germany), and the operational conditions were 650 rpm, per minute spin changing, and a temperature of 20 °C.

HPLC grade solvents and metallic oxides (palladium (II) acetate and titanium (IV) isopropoxide) were purchased from Sigma-Aldrich (Darmstadt, Germany).

Catechol (C_6_H_6_O_2_, ≥ 99.5% purity) and methyldopa (C_10_H_13_NO_4_, 99% purity) were purchased from Sigma-Aldrich (Germany) and standard solutions of these compounds were prepared as 1 mmol/L stock solutions.

Pharmaceutical formulation (containing methyldopa) analyzed in this work was purchased from a local drugstore, Goiânia-Go, Brazil.

### 4.2. Synthesis and Characterization of Graphite Powder

The graphite powder (1.2 g) was immersed in two different solutions of 15 mL ethanol and 15 mL acetone, respectively. They were mixed and heated for 10 min at 30 °C (Grupo Selecta Agimatic-N, Spain). After that, to achieve the 5% metallic solutions, the graphite powder was mixed with 0.13 mL titanium(IV)isopropoxide and 0.13 g palladium(II)acetate in 5 mL of ethanol and acetone, respectively. Thereafter, each of these metallic solutions was added drop by drop during two hours at the carbon solutions, under constant mixing and heating constantly at 30 °C.

The Ti(IV) and Pd(II) nanostructured powders were recovered using a rotary evaporator (Heidolph Rotary Evaporator, Laborota 4000, Germany). The rotation was constant at 100 rpm; at 60 °C and 40 °C for acetone and ethanol solutions, respectively. Finally, powders were dried in a vacuum desiccator at 70 °C (Selecta Vacuo-temp heated vacuum desiccator – AAR 3356, Barcelona - Spain).

Scanning Electron Microscopy (SEM) experiments for graphite powder morphology characterization were conducted on a JSM-6610 model instrument (JEOL Ltd., Musashino, Akishima, Tokyo, Japan). The magnification range was 1000 to 15,000, with an accelerating voltage of 15.0 kV. The elemental analysis of electrodes was performed by means of energy-dispersive X-ray (EDX) analysis on the JSM-6610 model instrument.

### 4.3. Preparation of Sensors

CPEs were prepared by mixing rigorously 100 mg of graphite powder and 100 mg of modified graphite powder of PdO (PdO@CPE) or TiO_2_ (TiO_2_@CPE) in a mortar, with 30 mg of mineral oil (as agglutinating agent). Thereafter, 30 mg of mineral oil was then added to the dried powder and thoroughly mixed in a mortar with pestle, leading to a homogeneous carbon paste. Appropriate portions of the agglutinated carbon pastes were used to fill the cavity (2 mm diameter and 0.5 mm depth) of the supporting electrode set-up [13,16,21].

### 4.4. Electrochemical Assays

Electrochemical impedance spectroscopy (EIS) and differential pulse voltammetry (DPV) measurements were performed with a potentiostat/galvanostat PGSTAT^®^ model 204 with module FRA32M (Autolab Electrochemical Instruments, Utrecht, The Netherlands) integrated with NOVA 2.1^®^ software. Both measurements were performed in a 5 mL one-compartment electrochemical cell, with a three-electrode system consisting of the newly composed CPE based sensors, Pt wire, and Ag/AgCl/KCl_sat_ (both purchased from Lab solutions, São Paulo, Brazil), representing the working, counter, and reference electrode, respectively. The carbon paste was mechanically renewed for every new assay [13,16,21].

EIS measurements were conducted in a solution containing 0.05 mol/L potassium ferrocyanide in 0.1 mol/L KCl over a frequency ranging from 0.01 Hz to 100 kHz at selected potentials for each sensor.

The experimental conditions for DPV measurements were: pulse amplitude 50 mV, pulse width 0.5 s, and scan rate 5 mV/s (both experimentally optimized) [13,14,15,16,17,18,19]. All experiments were done at room temperature (21 ± 2 °C) in triplicate (*n* = 3) and the main electrolyte solution used was phosphate buffer (PB) solution. The DP voltammograms were background-subtracted and baseline-corrected. Plots of the voltammetric curves for final presentation in this study were drawn using Origin Pro 8^®^ software (Northampton, MA, USA).

### 4.5. Real Sample Preparation

For pharmaceutical formulations analyses, some tablets containing methyldopa (labeled 250 mg per tablet) were completely grinded and dissolved in 0.05 mol/L HCl solution. These solutions were refrigerated during and after voltammetric analysis. 

## 5. Conclusions

The TiO_2_@CPE sensor developed in this study is a cheap, reliable, and useful strategy to detect methyldopa in pharmaceutical samples, since it presents excellent electrochemical behavior, which is nonetheless correlated to better electrodic applicabilities, and was able to detect methyldopa with a good linear range at pH = 5.0, between 10–180 μmol/L (Limit of Detection = 1 μmol/L). Moreover, our results suggest that TiO_2_@CPE may also promote analyte electro-oxidation, henceforth increasing method sensibility. Furthermore, the electrode herein investigated may be a valuable and cheap alternative to determine compounds such as methyldopa in drug formulae, and moreover shed light on the use of low-cost electrodic matrices in pharmaceutical analysis. 

## Figures and Tables

**Table 1:** Energy-dispersive X-ray (EDX) elementary results for comparison between unmodified graphite powder and nano-modified graphite powder.

Material	Analyses EDX					
	% Ti	% Pd	% C	% O	% Si	% Al
Carbon graphite (C)	-	-	76.10	15.19	6.16	2.54
TiO2@C	4.00 2.26	-	70.33 73.26	18.14 18.61	5.32 4.10	2.21 1.77
PdO@C	-	10.63 13.70	58.46 52.42	14.36 14.38	12.16 14.10	4.39 5.40

**Table 2:** Randles equivalent circuit elements for each electrode.

	Electrodes		
Circuit Elements	CPE	PdO@CPE	TiO_2_@CPE
*R_s_*	610 Ω	424 Ω	176 Ω
*R_p_*	4.57 kΩ	1480 kΩ	2.87 kΩ
*C*	0.064 µF	0.514 µF	3.65 µF
*Y*	5.69 µMho.s^1/2^	3.66 µMho.s^1/2^	162 µMho.s^1/2^

CPE = carbon paste electrode.

**Table 3:** Comparison of results obtained for methyldopa detection with different electrodes and the work done in this study.

Electrode	Methods	LOD (µmol/L)	Linear Range (µmol/L)	Reference
TiO_2_@CPE	DPV_reduction_	1.00	10–180	This work
GCE-TGA-capped-CdSe@Ag_2_Se	DPV_oxidation_	0.04	0.09–60	[1]
5ADB-CTNs-CPE	SWV	0.048	0.1–210	[3]
NiO-IL-CPE	SWV	0.06	0.1–700	[2]
GCE-NiFe_2_O_4_-MWCNTs	DPV_oxidation_	0.08	0.5-900	[18]
GCE/Lacc	DPV_reduction_	4.5	25-100	[19]
GCE-MWCNTs	SWV	0.001	0.005-0.388	[20]

CPE = carbon paste electrode; GCE = glassy carbon electrode; TGA = thioglycolic acid; 5AEB = 5-amino-20-ethyl-biphenyl-2-ol; CNTs = carbon nanotubes; SWV = square wave voltammetry; IL = ionic liquid, MWCNTs = carboxylated multiwalled carbon nanotubes; Lacc = enzyme laccase; DPV = differential pulse voltammetry; LOD = limit of detection.

**Table 4:** Results obtained for the determination of methyldopa in standard and standard plus placebo solutions.

Method	Added Methyldopa (mg)	Recovered Methyldopa (mg)	Mean Recovery (%) ± RSD ^a^
Pure standard	250	250.72	100.29% (± 3.94)
Standard plus placebo ^b^	250	251.15	100.46% (± 4.33)

^a.^ RSD: Relative standard deviation of three replicate determinations, average of three replicate determinations. ^b^ placebo composed by tenfold of starch, zinc stearate, aerosol®, and carboxymethyl-cellulose.

**Table 5:** Results obtained for the determination of methyldopa in commercial pharmaceutical formulations (tablets) using the proposed DPV method and official method [25].

Method	Sample	Labeled Concentration (mg/tablet)	Experimental Concentration (mg)	Mean Recovery (%) ± RSD ^a^
Official Method	tablet	250	250.62	100.25% (± 4.14)
DPV at TiO_2_@CP	tablet	250	251.02	100.41% (± 4.64)

^a^ SD: Standard deviation of three replicate determinations, average of three replicate determinations.
